# The Intersection between Sex Work and Reproductive Health in Northern Karnataka, India: Identifying Gaps and Opportunities in the Context of HIV Prevention

**DOI:** 10.1155/2012/842576

**Published:** 2012-12-30

**Authors:** Marissa Becker, Satyanarayana Ramanaik, Shiva Halli, James F. Blanchard, T. Raghavendra, Parinita Bhattacharjee, Stephen Moses, Lisa Avery, Sharmistha Mishra

**Affiliations:** ^1^Center for Global Public Health, University of Manitoba, R070 Med Rehab Building, 771 McDermot Avenue, Winnipeg, MB, Canada R3E 0T6; ^2^Karnataka Health Promotion Trust, Rajajinagar, Bangalore, Karnataka 560044, India; ^3^Department of Infectious Disease Epidemiology, Imperial College London, London SW 7 2A2, UK; ^4^Department of Medicine, St. Michael's Hospital, University of Toronto, Toronto, Canada ON, M5B 1W8

## Abstract

*Objective*. To examine the reproductive health practices of female sex workers (FSWs) in the context of an HIV prevention program in Karnataka, India. *Methods*. Data obtained from a survey of 1,011 FSWs registered with an HIV prevention program. We examined reproductive health indicators, and performed multivariate logistic regression among primiparous FSWs to assess sex work during pregnancy and antenatal HIV testing. *Results*. Among primiparous FSWs (*N* = 251), 92.0% continued sex work during pregnancy, and 55.4% received antenatal HIV testing. A longer duration in sex work (AOR 2.7, 95% CI: 1.0–7.5), rural residence (AOR 3.3, 95% CI: 1.2–8.9), and antenatal HIV testing (AOR 6.3, 95% CI: 2.0–20.1) were associated with continued sex work during pregnancy. Older FSWs (age >25 years, AOR 0.12, 95% CI: 0.05–0.33), who delivered at home (AOR 0.14, 95% CI: 0.09–0.34), were least likely to receive antenatal HIV testing. Antenatal HIV testing was associated with awareness of methods to prevent vertical HIV transmission (AOR 3.9, 95% CI: 1.9–14.1). *Conclusions*. Antenatal HIV testing remains low in the context of ongoing sex work during pregnancy. Existing HIV prevention programs are well positioned to immediately integrate reproductive health care with HIV interventions targeted to FSWs.

## 1. Introduction

There is growing recognition of the need to strengthen linkages between reproductive health and HIV prevention services for female sex workers (FSWs) [1–6]. This is especially important in regions where HIV is predominantly spread through heterosexual and vertical transmission, and where unprotected sex between FSWs and their clients are key drivers of the HIV epidemic [7]. To date, research and intervention programs have primarily focused on the burden of, and vulnerability to, sexually transmitted infections (STIs) and HIV in FSWs [8–10]. Meeting FSWs' need for contraception and antenatal care alongside HIV/STI prevention is critical given their high rates of pregnancy, often unintended, [11–13] as well as high rates of HIV and STIs. 

A small body of literature on the reproductive health of FSWs in developing countries suggests that many FSWs rely solely on condoms for birth control [4, 6, 13], and experience a high rate of unintended pregnancies and voluntary abortions [1, 4, 6, 11, 14–16]. For example, 25% of FSWs in Goa, India reported at least one voluntary abortion [1]. While previous studies have raised concerns about unmet contraceptive needs of FSWs [4, 6], there remains a dearth of literature surrounding the practice of sex work and HIV testing during pregnancy. High prevalence and incidence of HIV among FSWs suggests that antenatal HIV testing in FSWs who intend to carry to term (followed by the provision of antiretrovirals for prevention of vertical transmission and maternal HIV treatment, as appropriate) should be a priority. Understanding the reproductive health of FSWs, particularly sex work during pregnancy, will be key to integrating HIV prevention and reproductive health programs.

The state of Karnataka, in south India, is experiencing an HIV epidemic driven by sex work [17, 18]. With an HIV prevalence of 1.0% among females attending antenatal clinics in 2008, the state is home to 49,170 women of reproductive age living with HIV/AIDS [19]. By 2009, the HIV prevalence among FSWs in Karnataka was 16.4% [8, 20]. FSWs living in some of the northern districts are at highest risk of HIV (34.2% HIV prevalence) [8]. After characterizing the HIV/STI risk and vulnerabilities faced by FSWs, program scientists were able to design and implement effective targeted HIV/STI prevention programs in the state [8, 21, 22]. By 2009, over 84% of FSWs in Karnataka reported using a condom during their last commercial sex act [20]. However, programs are currently implemented as vertical silos. There is little overlap between reproductive health services (family planning and prevention of mother to child transmission [PMTCT] [23]) designed for the overall female population and targeted HIV/STI prevention services designed for FSWs [24]. As HIV prevention programs in India transition from nongovernmental organizations (NGOs) to local ministries of health [25], there is an opportunity to strengthen linkages between reproductive health care and targeted HIV prevention. 

In northern Karnataka, transactional sex is practiced in the context of traditional (*Devadasi*) and nontraditional sex work [26]. Details of the *Devadasi* tradition have been previously described [27, 28]. In brief, *Devadasis* comprise women who have been ritually dedicated (often by their families) into sex work following menarche [27, 28]. While *Devadasis* traditionally do not marry, long-term partnerships are common [29]. Furthermore, evidence suggests that *Devadasis* have a 2-fold higher HIV prevalence than nontraditional FSWs [8]. The practice of sex work is different between these two typologies of sex work: *Devadasis* formally enter sex work at an earlier age, are more likely to temporarily migrate outside the state for sex work (in addition to local sex work), and are more likely to practice sex work from their homes (home-based sex work [26]). Nontraditional sex work is primarily brothel-based, street-based, or conducted in lodges and hotels [26].

To address a gap in knowledge surrounding the reproductive health of traditional and nontraditional FSWs in northern Karnataka, we analyzed data from a cross-sectional survey of FSWs who recently initiated sex work and were registered in the local HIV/STI prevention program. Our aim was to examine the reproductive health practices of FSWs, with a focus on sex work and HIV testing during pregnancy. 

## 2. Methods

### 2.1. Study Setting and Population

We used secondary data from a behavioural cross-sectional survey of FSWs who formally entered sex work within five years prior to data collection. The primary objective of the survey was to examine the behavioral patterns of FSWs early in their sex work career. The survey was conducted in three districts of northern Karnataka between January and June 2011: Belgaum, Bagalkot, and Bijapur. Targeted HIV/STI interventions for FSWs have been implemented in the region since 2004, and fall under the remit of a large-scale HIV prevention program (the National AIDS Control Organization [24] and* Avahan*, the India AIDS initiative of the Bill & Melinda Gates Foundation [25]). An estimated 20,493 women were practicing sex work across the three districts in 2010, of whom 38.3% were *Devadasis *[30]. FSWs are registered with the program at first contact with a peer-educator or outreach worker who actively seek and identify FSWs on an ongoing basis. At the time of sampling, 97% of enumerated FSWs had been registered with the local program [30]. For the survey, the sample frame included 11,028 registered FSWs who were currently engaged in sex work and began sex work in the previous five years. 

The survey used conventional cluster sampling with probability proportional to size of the enumerated FSW population in each village. A total of 1,500 FSWs were sampled. If FSWs were not at their place of residence during the first study visit, two repeat visits were made as well as contact through community researchers (peers) to arrange interviews. Trained interviewers obtained written informed consent and conducted face-to-face interviews with a structured questionnaire in the local language (*Kannada*). At this stage, interviewers independently confirmed study eligibility: FSWs who had received money or gifts in exchange for sex in the past year and reported entering sex work after December 2005 were included in the survey. 

### 2.2. Measures

The questionnaire collected self-reported data on sociodemographic characteristics, sexual behaviour, and reproductive health practices. The questionnaire was pilot tested prior to data collection. Contraceptive methods reflect current use, and respondents could list more than one method for birth control. Traditional sex workers were defined as FSWs who identified as *Devadasis*. Nontraditional FSWs (i.e., non-*Devadasis*) included street-based, home-based, or brothel-based FSWs. FSWs were classified as mobile if they engaged in sex work outside their village or city of residence (with or without sex work at home). PMTCT awareness and knowledge were assessed by asking the following two questions: “Are there ways to prevent a baby from acquiring HIV infection when the mother is HIV positive?”, and if the answer was “yes,” to list all approaches to preventing vertical transmission. Awareness (“yes” versus either “no” or “do not know”) was based on the first question. Adequate knowledge was scored as “yes” if responses to the second question included at least one of the following: medication, antiretroviral treatment/drugs, medical treatment. The questionnaire did not include self-reported HIV status. However, data on registration in antiretroviral treatment centers (linkage to HIV care after a positive HIV test) was available.

### 2.3. Analysis

We reported descriptive statistics for demographic characteristics and reproductive health indicators for all study participants. In the questionnaire, information surrounding sex work and HIV testing during pregnancy was elicited for “any pregnancy,” and therefore could not be reliably inferred for a unique pregnancy among multiparous FSWs. Hence, to enable a precise examination of sex work and HIV testing during pregnancy, we restricted the analysis to the following subset of FSWs: primiparous respondents whose pregnancy took place after formal entry into sex work. Questions surrounding HIV tests were asked separately for tests performed as an “antenatal HIV test while pregnant” versus HIV testing outside of a known pregnancy. We used univariate and multivariate logistic regression to characterize primiparous FSWs for the following outcomes: (a) practiced sex work during pregnancy and (b) received an HIV test during the pregnancy. To profile primiparous FSWs who received antenatal HIV testing, the multivariate analysis was restricted to FSWs who did not experience a spontaneous or voluntary abortion. Only variables significant at the 5% alpha-level on univariate analysis were considered in the multivariate analysis. The final multivariate model reflected a best fit, obtained using forward stepwise regression and the likelihood ratio test. Analyses were unweighted and adjusted for intracluster correlation to account for the sampling design. The analyses were performed in Stata 11 (Statacorp).

### 2.4. Ethical Approval

Institutional review boards at the University of Manitoba, Canada and St. John's Medical College and Hospital, Bangalore, India, provided ethical approval.

## 3. Results

### 3.1. Demographic and Reproductive Health Indicators

Of the 1,500 FSWs invited to participate, 1,182 (78.8%) consented to the interview, and among them, 1,011 met eligibility criteria.  Table 1 describes the socio-demographic characteristics and reproductive health indicators of all participants. The median age of participants was 22 years (range, 15 to 32). The majority of FSWs were illiterate, resided in rural villages, and had practiced sex work for more than one year. A quarter (24.3%) of the FSWs identified as *Devadasi *and about one third of the participants reported travelling outside of their place of residence to conduct sex work (in addition to local sex work). Over 90% of participants had been tested for HIV at least once (not including antenatal HIV tests).

Most FSWs (83.7%) reported at least one pregnancy, while 7.7% had undergone at least one voluntary abortion and 18.9% reported at least one pregnancy loss (spontaneous abortion or stillbirth). Nearly half of FSWs (45.2%) had undergone tubal ligation. While contraceptive use appeared to be reasonably high (87.3% of FSWs were using at least one form of contraception), 41.4% of respondents used condoms as their only method of contraception. Only 23.2% of FSWs were aware of methods to prevent vertical transmission of HIV, and 6.4% demonstrated adequate knowledge of PMTCT tools (Table 1).

### 3.2. Pregnancy, Sex Work, and HIV

Table 2 depicts factors associated with continuation versus cessation of sex work during pregnancy among primiparous FSWs. None of the primiparous respondents were pregnant at the time of the survey (based on self-report). In this subset of FSWs, the pregnancy occurred after formal entry into sex work. Overall, 92.0% (95% CI: 87.7–94.9) of primiparous FSWs continued to sell sex after they became aware of their pregnancy (Table 2). While only 6.8% (95% CI: 4.1–11.0) engaged in sex work beyond six months into the pregnancy, 64.5% (95% CI: 58.1–70.5) continued until approximately the third month of pregnancy, and 20.7% (95% CI: 14.5–28.7) continued until the third trimester. Among primiparous FSWs, a longer duration in sex work (adjusted odds ratio (AOR) 2.7, 95% CI: 1.0–7.5), rural residence (AOR 3.3, 95% CI: 1.2–8.9), and antenatal HIV testing (AOR 6.3, 95% CI: 2.0–20.1) were independently associated with continued sex work during pregnancy.

Among primiparous FSWs, 44.2% (95% CI: 36.8-51.8) of FSWs delivered at home (excluding those who underwent a voluntary abortion or experienced a spontaneous abortion). Among primiparous FSWs, only 24.7% (95% CI: 18.5–32.2) were aware of methods to prevent vertical transmission of HIV. Among primiparous FSWs who were aware of PMTCT (*N* = 62), 30.7% (95% CI: 19.0–45.4) could demonstrate adequate knowledge of PMTCT tools.

Among primiparous FSWs, 55.4% (95% CI: 47.8–62.8) received antenatal HIV testing (Table 3), although 93.2% (95% CI: 89.5–95.7) had received at least one HIV test outside of pregnancy. Among primiparous FSWs who received antenatal HIV testing, 52.5% (95% CI: 43.8–61.1) were tested at government voluntary testing and counselling centers (VCT), and the remainder were tested in private clinics. Of note, 40.3% (95% CI: 31.6–49.6) of primiparous FSWs received an antenatal HIV test due to referral by staff in the local HIV prevention program. 

Among primiparous FSWs who did not experience a spontaneous or voluntary abortion (Table 3), antenatal HIV testing was independently associated with awareness of PMTCT (AOR 3.9, 95% CI: 1.9–14.1) and continued sex work during pregnancy (AOR 3.9, 95% CI: 1.01–20.2). In this same subgroup, older FSWs (age >25 years, AOR 0.12, 95% CI: 0.05–0.33), who delivered at home (AOR 0.14, 95% CI: 0.09–0.34), were least likely to receive antenatal HIV testing. There was no association between antenatal HIV testing and registration in a pre-antiretroviral treatment center prior to or at the time of pregnancy (Table 3).

## 4. Discussion

We documented considerable gaps in the reproductive health practices of FSWs new to sex work but registered with a targeted HIV prevention program. As in other resource-poor settings [1, 14–16], most FSWs had been pregnant at least once. While 92.0% of primiparous FSWs continued to practice sex work during pregnancy, only half received antenatal HIV testing, and 75.3% remained unaware of methods to prevent vertical HIV transmission. The findings have important implications for individual and public health in the region: among new FSWs, there may be an ongoing risk of vertical HIV transmission to newborns, especially in the setting of low antenatal HIV testing. The findings also suggest an immediate opportunity for integrating reproductive health services with targeted HIV prevention early in a FSWs' career, because most FSWs (including new entrants) are already registered with HIV prevention programs.

We found that antenatal HIV testing among new FSWs remains inadequate in northern Karnataka. Lack of HIV testing and lack of awareness of one's HIV status during pregnancy are barriers to the prevention of mother to child transmission of HIV. It is estimated that in India, 21% of pregnant women received antenatal HIV testing in 2009 [31], although estimates for Karnataka are not available. Although primiparous FSWs and a population of all pregnant women cannot be directly compared, low antenatal HIV testing among FSWs appears to mimic that of low antenatal HIV testing rates in the overall population in India [31]. Among FSWs, the low uptake of HIV testing during pregnancy is in stark contrast to high levels of overall HIV testing outside of pregnancy. Education about routine HIV testing has been an important part of the FSW program, although tests are provided by government voluntary and counselling centers and not the HIV/STI intervention program. However, education and awareness of vertical transmission falls under the purview of the general population interventions for HIV prevention (not the FSW program). We found that PMTCT messages and services are not reaching FSWs, even though FSWs incur the largest burden of HIV in the region. Older FSWs who delivered at home and were unaware of methods to prevent vertical transmission were least likely to receive antenatal HIV testing during their one pregnancy. Although we lacked data on family planning desires, this profile of unmet need excluded FSWs who experienced a spontaneous or voluntary abortion. Importantly, we found that among FSWs who received antenatal HIV testing, 40.3% were referred by staff at the local HIV prevention program. Systematic referral for antenatal HIV testing by program staff is a feasible strategy and offers an immediate opportunity to expand antenatal HIV testing and increase awareness of PMTCT among FSWs. By doing so, existing HIV prevention programs could begin to systematically integrate reproductive health services.

We also documented that most new FSWs continue sex work during pregnancy—suggesting a need for HIV retesting among FSWs who plan to carry to term. Although the findings are restricted to primiparous FSWs, ongoing high-risk sex during pregnancy places the mother and fetus at even greater risk of acquiring HIV (and certain STIs) [32]. At present, national guidelines [23] do not include antenatal HIV retesting for high-risk groups (i.e., FSWs). Recent evidence from the general population in Africa—where HIV prevalence among adult women is similar to HIV prevalence among FSWs in India—suggests that 1–3% of pregnant women who are initially HIV seronegative will seroconvert prior to delivery [33, 34]. In South Africa, single women were at highest risk of HIV sero-conversion during pregnancy [32]. Children born to women with incident (primary) HIV infection during pregnancy have a 2.3-fold higher risk of HIV infection compared to children born to women at later stages of maternal HIV infection [32]. As a result, HIV retesting during pregnancy is advocated—particularly for women at highest-risk for HIV [33, 35]. Further study is needed to estimate HIV incidence during pregnancy among FSWs in India. Nonetheless, based on our findings, we recommend that pregnant FSWs who plan to carry to term should be retested for HIV during pregnancy given ongoing exposure.

To our knowledge, this is the first study to examine continued sex work and HIV testing during pregnancy among FSWs. However, our study has a number of limitations. Chief among them is the limited generalizability to multiparous FSWs, due to the restricted subset analysis. Lack of data on self-reported HIV status could also overestimate the magnitude of the gap in antenatal HIV testing. HIV-infected FSWs aware of their HIV status prior to the first pregnancy may not have been tested again during pregnancy. However, registration in pre-antiretroviral treatment centers (reflecting linkage into HIV care after an HIV diagnosis) was not associated with lower antenatal HIV testing. Our analysis was limited by available data on reproductive health indicators, and as such, pregnancy-related information for each pregnancy among multiparous FSWs, family planning desires of FSWs, self-reported HIV status, and antenatal HIV retesting were not available. The study sample reflects a subset of FSWs new to sex work and already reached by the local HIV/STI intervention program. Although the program has achieved a high coverage of enumerated FSWs, our findings may not be generalizable to FSWs not reached by HIV/STI prevention services or women engaged in sex work for longer than five years. Misreporting and measurement bias could also affect our findings, as women may feel uncomfortable disclosing culturally sensitive information around abortions or other reproductive health practices. Further study is needed to quantify and characterize sex work during pregnancy, and antenatal HIV testing and retesting, stratified by family planning desire and HIV status. A prospective study to measure HIV incidence during pregnancy if feasible, as well as uptake and coverage of PMTCT services among FSWs, is warranted.

## 5. Conclusion

We uncovered a substantial unmet need for HIV-related antenatal care for FSWs new to sex work and registered in an HIV/STI prevention program. The findings call for improved education and awareness of PMTCT tools and expanded HIV testing and retesting during pregnancy among FSWs—all of which could be addressed by integrating targeted HIV/STI prevention and general reproductive health services. Existing HIV/STI prevention programs provide an immediate opportunity to address this underexamined issue in maternal health and the prevention of vertical transmission of HIV and STIs among FSWs, particularly as programs transition into the public health sector.

## Figures and Tables

**Table 1:** Demographic characteristics and reproductive health indicators among female sex workers (FSWs) in Karnataka, India (*N* = 1,011).

Participant characteristics	*N*	%	95% CI
Age ≤25	457	45.2	38.9, 51.7
Unable to read or write	713	70.5	66.8, 74.0
Duration in sex work ≤1 year	171	16.9	14.1, 20.2
*Devadasi *	246	24.3	18.3, 31.7
Mobile*	359	35.5	30.5, 40.9
Rural	681	67.4	55.5, 77.3
Ever tested for HIV (outside of pregnancy)	953	94.3	92.4, 95.7
Ever had a long-term (noncommercial) partner	928	91.8	88.1, 94.4
Registered in pre-antiretroviral treatment centers (linked to HIV care after a positive HIV test)	68	6.7	4.9, 9.2

Reproductive health indicators			

Ever pregnant	846	83.7	80.6, 86.4
Number of living children			
None	253	25.0	21.1, 29.4
1–3	667	66.0	62.1, 69.7
>3	91	9.0	6.6, 12.2
Current contraceptive use**			
None	128	12.7	10.4, 15.4
Oral hormone contraceptives	5	0.5	0.2, 1.2
Intrauterine device	3	0.3	0.0, 1.3
Condoms alone	418	41.4	35.8, 47.2
Tubal ligation	457	45.2	39.5, 51.1

Median number of pregnancies (range)	2 (0,9)	—	—

Number of pregnancies			
Nulliparous	165	16.3	13.6, 19.5
Primiparous	251	24.8	21.5, 28.5
Multiparous	659	58.9	53.8, 63.7
≥1 pregnancy loss (spontaneous abortion or stillbirth)	191	18.9	16.2, 21.9
≥1 voluntary abortion	78	7.7	4.9, 12.0
Aware of methods to prevent mother to child transmission of HIV	235	23.2	20.0, 26.9
Adequate knowledge of methods to prevent mother to child transmission of HIV	65	6.4	5.0, 8.2

*FSWs who also travel to a village or city outside their place of residence to conduct sex work. **The following methods (oral hormone contraceptives, intrauterine device, and tubal ligation) could include the use of condoms for contraception.

**Table 2:** Factors associated with ongoing sex work during pregnancy among primiparous female sex workers (*N* = 251).

	*N*	Continued sex work during pregnancy after becoming aware of pregnancy (*N* = 231)				
		Row %	Univariate analysis		Multivariate analysis***	
			Crude OR (95% CI)	*P* value	Adjusted OR (95% CI)	*P* value
Age-group						
≤25 >25	154 97	93.5 89.7	Ref 0.60 (0.24, 1.5)	0.3	—	—
*Devadasi* status						
No Yes	158 93	89.9 95.7	Ref 2.5 (0.81, 7.7)	0.1	—	—
Literacy						
Literate Illiterate	178 73	92.7 90.4	Ref 0.74 (0.28, 1.9)	0.5	—	—
Duration in sex work						
≤1 year >1 year	49 202	83.7 94.1	Ref 3.1 (1.2, 8.0)	0.02	Ref 2.7 (1.0, 7.5)	0.05
Long-term partner at time of pregnancy						
No Yes	38 213	91.7 94.6	Ref 1.6 (0.74, 3.3)	0.2	—	—
Mobile*						
No Yes	145 106	92.4 91.5	Ref 0.88 (0.35, 2.2)	0.8	—	—
Place of residence (%)						
Urban Rural	89 162	86.5 95.1	Ref 3.0 (1.2, 7.6)	0.02	Ref 3.3 (1.2, 8.9)	0.02
Received at least one HIV test during the pregnancy						
No Yes	112 139	85.7 97.0	Ref 5.6 (1.8, 17.4)	0.003	Ref 6.3 (2.0, 20.1)	0.002
Voluntary abortion during the pregnancy						
No Yes	209 42	92.8 88.0	Ref 0.57 (0.20, 1.7)	0.3	—	—
Experienced pregnancy loss (spontaneous abortion or stillbirth) during the pregnancy						
No Yes	194 57	93.8 84.0	Ref 0.40 (0.16, 1.0)	0.06	—	—
Place of delivery (*N* = 197**)						
Health-care facility Home	110 87	98.2 95.4	Ref 0.38 (0.07, 2.1)	0.3	—	—
Aware of methods to prevent mother to child transmission of HIV						
No Yes	189 62	91.5 93.6	Ref 1.3 (0.43, 4.2)	0.6	—	—

*FSWs who also travel to a village or city outside their place of residence to conduct sex work. **Includes stillbirth. ***Multivariate analysis includes primiparous FSWs who experienced a miscarriage, or received a voluntary abortion. Final multivariate model includes the following variables: duration in sex work, place of residence, antenatal HIV testing.

**Table 3:** Factors associated with HIV testing during pregnancy, among primiparous female sex workers (*N* = 251).

	*N*	Received HIV test during pregnancy (*N* = 139)				
		Row %	Univariate analysis		Multivariate analysis***	
			Crude OR (95% CI)	*P*-value	Adjusted OR (95% CI)	*P*-value
Age-group						
≤25 >25	154 97	68.2 35.1	Ref 0.25 (0.15, 0.43)	<0.001	Ref 0.12 (0.05, 0.33)	<0.001
*Devadasi* status						
No Yes	158 93	50.0 64.5	Ref 1.8 (1.1, 3.1)	0.03	—	—
Literacy						
Literate Illiterate	178 73	53.4 60.3	Ref 1.3 (0.76, 2.3)	0.3	—	—
Duration in sex work						
≤1 year >1 year	49 202	53.1 55.9	Ref 1.1 (0.60, 2.1)	0.7	—	—
Ongoing sex work during pregnancy						
No Yes	20 231	20.0 58.4	Ref 5.6 (1.8, 17.4)	0.003	Ref 3.1 (1.01, 20.2)	0.04
Long-term partner at time of pregnancy						
No Yes	38 213	73.7 52.1	Ref 0.39 (0.18, 0.84)	0.03	—	—
Mobile*						
No Yes	145 106	57.2 52.8	Ref 0.84 (0.51, 1.4)	0.5	—	—
Place of residence (%)						
Urban Rural	89 162	60.7 52.5	Ref 0.72 (0.43, 1.2)	0.2	—	—
Voluntary abortion during the pregnancy						
No Yes	209 42	57.9 42.9	Ref 0.55 (0.28, 1.1)	0.08	—	—
Experienced pregnancy loss (spontaneous abortion or stillbirth) during the pregnancy						
No Yes	194 57	58.3 45.6	Ref 0.60 (0.33, 1.1)	0.09	—	—
Place of delivery (*N* = 197**)						
Health-care facility Home	110 87	77.3 40.2	Ref 0.20 (0.11, 0.37)	<0.001	Ref 0.14 (0.09, 0.34)	<0.001
Aware of methods to prevent mother to child transmission of HIV						
No Yes	189 62	47.6 79.0	Ref 4.1 (2.1, 8.1)	<0.001	Ref 3.9 (1.9, 14.1)	<0.001
Registered in pre-antiretroviral treatment centers (linked to HIV care after a positive HIV test)						
No Yes	237 14	55.3 57.1	Ref 1.1 (0.36, 3.2)	0.9	—	—

*FSWs who also travel to a village or city outside their place of residence to conduct sex work. **Includes stillbirth. ***Multivariate analysis excludes primiparous FSWs who experienced a miscarriage or received a voluntary abortion. Final multivariate model includes the following variables: age, continued sex work during pregnancy, place of delivery, and awareness of methods to prevent mother to child transmission of HIV. Registration in pre-antiretroviral treatment centers refers to registration prior to or at time of the pregnancy.
